# Magnetic resonance and dopamine transporter imaging for the diagnosis of Parkinson´s disease: a narrative review

**DOI:** 10.1590/0004-282X-ANP-2022-S130

**Published:** 2022-08-12

**Authors:** Rafael Tomio Vicentini Otani, Joyce Yuri Silvestre Yamamoto, Douglas Mendes Nunes, Mônica Santoro Haddad, Jacy Bezerra Parmera

**Affiliations:** 1 Universidade de São Paulo, Faculdade de Medicina, Hospital das Clínicas, Departamento de Neurologia, São Paulo SP, Brazil.; 2 Universidade de São Paulo, Faculdade de Medicina, Hospital das Clínicas, Departmento de Radiologia e Oncologia, Instituto de Radiologia, São Paulo SP, Brazil.

**Keywords:** Parkinson Disease, Parkinsonian Disorders, Diffusion Tensor Imaging, Single Photon Emission Computed Tomography Computed Tomography, Melanins, Magnetic Resonance Imaging, Diffusion Magnetic Resonance Imaging, Doença de Parkinson, Transtornos Parkinsonianos, Imagem de Tensor de Difusão, Melaninas, Imageamento por Ressonância Magnética, Imagem de Difusão por Ressonância Magnética

## Abstract

**Background:**

the diagnosis of Parkinson's disease (PD) can be challenging, especially in the early stages, albeit its updated and validated clinical criteria. Recent developments on neuroimaging in PD, altogether with its consolidated role of excluding secondary and other neurodegenerative causes of parkinsonism, provide more confidence in the diagnosis across the different stages of the disease. This review highlights current knowledge and major recent advances in magnetic resonance and dopamine transporter imaging in aiding PD diagnosis.

**Objective:**

This study aims to review current knowledge about the role of magnetic resonance imaging and neuroimaging of the dopamine transporter in diagnosing Parkinson's disease.

**Methods:**

We performed a non-systematic literature review through the PubMed database, using the keywords "Parkinson", “magnetic resonance imaging”, “diffusion tensor”, “diffusion-weighted”, “neuromelanin”, “nigrosome-1”, “single-photon emission computed tomography”, “dopamine transporter imaging”. The search was restricted to articles written in English, published between January 2010 and February 2022.

**Results:**

The diagnosis of Parkinson's disease remains a clinical diagnosis. However, new neuroimaging biomarkers hold promise for increased diagnostic accuracy, especially in earlier stages of the disease.

**Conclusion:**

Future validation of new imaging biomarkers bring the expectation of an increased neuroimaging role in the diagnosis of PD in the following years.

## RESUMO

**Antecedentes:**

O diagnóstico da doença de Parkinson (DP) pode ser desafiador, principalmente nas fases iniciais da doença, embora tenha critérios clínicos atualizados e validados. Os avanços recentes em neuroimagem na DP, além do seu papel já consolidado de excluir causas secundárias e outras causas neurodegenerativas de parkinsonismo, tem contribuído para uma maior confiabilidade no diagnóstico em diferentes estágios da doença. Nesta revisão, nós destacamos os principais avanços de ressonância magnética e imagem do transportador de dopamina em auxiliar o diagnóstico de DP.

**Objetivo:**

realizar uma revisão acerca do conhecimento atual sobre o papel da ressonância magnética e imagem do transportador de dopamina no diagnóstico de doença de Parkinson.

**Método:**

Realizamos uma revisão não sistemática da literatura através da base de dados PubMed, utilizando as palavras-chave "Parkinson", “magnetic resonance imaging”, “diffusion tensor”, “diffusion-weighted”, “neuromelanin”, “nigrosome-1”, “single-photon emission computed tomography”, “dopamine transporter imaging”. A busca foi restrita a artigos escritos em inglês, publicados entre janeiro de 2010 e fevereiro de 2022.

**Resultados:**

O diagnóstico de doença de Parkinson continua sendo um diagnóstico clínico, contudo, novos biomarcadores de neuroimagem são promissores para o aumento da acurácia diagnóstica, especialmente em fases mais precoces da doença.

**Conclusão:**

A validação futura de novos biomarcadores de imagem traz a expectativa de um maior papel da neuroimagem no diagnóstico de doença de Parkinson nos próximos anos.

## INTRODUCTION

Parkinson's disease (PD) represents the most common etiology of parkinsonism and the second most common neurodegenerative disease, with an estimated global prevalence of more than 9 million affected individuals^1^ ^-^ ^3^. Driven mainly by aging and additional factors such as increasing industrialization and declining smoking rates, this number is expected to rise to over 17 million by 2040^4^.

In recent years, there has been a significant advance in diagnosing PD, with novel clinical diagnostic criteria and research criteria for the prodromal disease stage, both proposed by the Movement Disorders Society (MDS)^5^ ^,^ ^6^. Despite these updated criteria, clinical diagnosis can often still be challenging, especially in earlier stages of the disease and if performed by nonexperts^7^ ^-^ ^9^. A previous clinicopathologic study by Hughes et al., which analyzed over 100 clinically diagnosed patients with PD, showed a relevant misdiagnosis rate of 10%^10^. Moreover, Adler et al. demonstrated, among patients clinically diagnosed with PD who underwent neuropathological examination, only 53% accuracy for a clinical diagnosis of PD in an early disease stage with less than five years duration^8^.

Recent developments of new neuroimaging techniques have been made possible with the emergence of high-field MR magnets, more sophisticated head coils, and improved MRI sequences. Neuroimaging in PD has expanded its role from just excluding secondary causes of parkinsonism to the appearance of new biomarkers that can aid in diagnosis across different stages of the disease, as well as assisting in its differential diagnosis with atypical parkinsonisms (AP), non-neurodegenerative causes of parkinsonism or even other movement disorders, such as essential tremor or functional movement disorders^2^ ^,^ ^11^.

The present study describes and critically reviews the current knowledge and most striking advances in MRI and dopamine transporter neuroimaging responsible for this role shift.

## SEARCH STRATEGY

We performed a non-systematic literature review through the PubMed database, using the disease-specific keyword "Parkinson", together with one of the modality-specific keywords: “magnetic resonance imaging”, “diffusion tensor”, “diffusion-weighted”, “neuromelanin”, “nigrosome-1”, “single-photon emission computed tomography”, “dopamine transporter imaging”. The search was restricted to articles written in English and published between January 2010 and February 2022. All abstracts were screened for relevance, and the most pertinent articles were then read and discussed.

## STRUCTURAL IMAGING IN T1/T2 MRI

**Figure 1.**
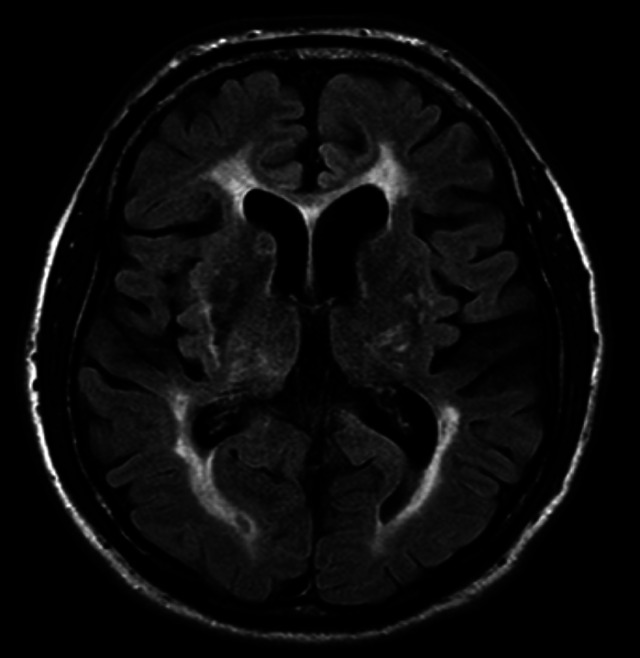
Vascular parkinsonism. Axial FLAIR MRI shows hyperintense foci involving the basal ganglia, thalamus, periventricular and subcortical white matter related to chronic small vessel ischemic disease**.**

In the early stages of PD, structural changes on conventional MRI are usually minimal or absent^12^. Although not essential for the clinical diagnosis, MRI should be requested at least one time during the disease course with two main objectives. The first is the exclusion of secondary causes of parkinsonism in the conventional sequences of T1 and T2, such as lesions with mass effect, demyelinating lesions, vascular alterations (Figure 1), normal pressure hydrocephalus, signs of deposit of metals (copper, iron, and manganese), and signs of traumatic brain injury^9^ ^,^ ^12^. The second is the search for imaging signs suggestive of AP.

AP comprises a group of less common and pathologically distinct disorders than PD, sharing their neurodegenerative condition and a parkinsonian syndrome as a clinical hallmark. From a neuropathological perspective, they can be divided, in a simplified way, into tauopathies, which comprises Progressive Supranuclear Palsy (PSP) and Corticobasal Degeneration (CBD), and synucleinopathies, which comprises Multiple System Atrophy (MSA) and Dementia with Lewy Bodies (DLB)^13^. Although these disorders tend to have a poor dopaminergic response and eventually manifest other signs and symptoms that can be distinguished from PD, these features may not be present early in the disease, and the differential diagnosis among these entities is challenging. In turn, T1/T2 structural MRI can help identify neuroimaging biomarkers that support the diagnosis of atypical parkinsonisms, with limited sensitivity and reasonable specificity.

PSP is clinically manifested by symmetric parkinsonism, supranuclear vertical gaze palsy, and early gait instability. Radiologically, the hallmark is midbrain area reduction leading to the visual identification of the “hummingbird sign” on the sagittal plane (specificity 99%, sensitivity 50%) and the “morning glory sign” on the axial plane (specificity 97%, sensitivity 37%); in addition to superior cerebellar peduncles (SCP) size reduction in the coronal plane^14^ ^,^ ^15^. Additionally, the magnetic resonance parkinsonism index (MRPI), calculated through the measurement of the ratios of the pons to midbrain area and middle cerebellar peduncle (MCP) to SCP widths, has shown high sensitivity and specificity for distinguishing PSP from PD, multiple system atrophy- parkinsonian type (MSA-P) and healthy controls^16^ ^,^ ^17^.

CBD is clinically characterized by asymmetric parkinsonism, often accompanied by dystonia, myoclonus, and cortical deficits. Structural MRI may demonstrate frontoparietal cortical atrophy contralateral to the most affected^18^. MSA is clinically characterized by various combinations of autonomic failure, parkinsonism, and ataxia. In MSA-P, bilateral T2/FLAIR hyperintense rim lining the dorsolateral borders of the putamen (“putaminal rim” sign), T2 putaminal hypointensity, and T1 atrophy of the putamen, cerebellum, pons, and MCP can be found. Regarding the cerebellar-predominant type (MSA-C), T2/FLAIR cruciform pontine hyperintensity known as “hot cross bun” sign (specificity 100%, sensitivity 58%), T2 MCP hyperintensity, and T1 atrophy of the putamen and MCP can be observed^19^ ^,^ ^20^. 

**Figure 2.**
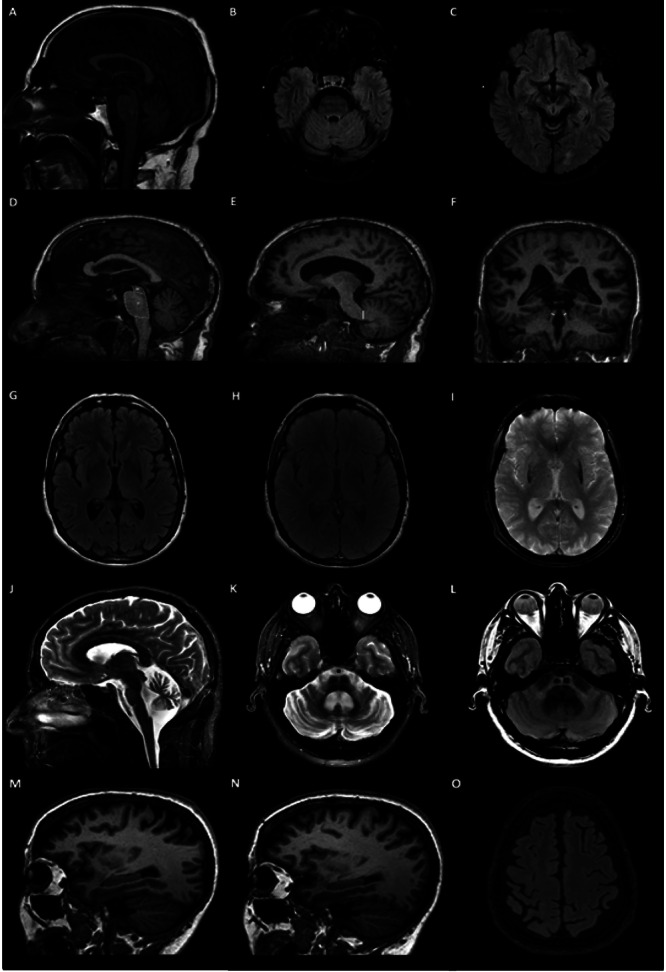
Atypical parkinsonism. Progressive supranuclear palsy. Midsagittal T1-weighted MRI (A) shows the “hummingbird sign”, result of selective atrophy of the midbrain tegmentum, with flattening or concave outline to the superior aspect of the midbrain, and relative pontine preservation. Axial FLAIR-weighted MRI shows SCP atrophy (B), reduction of anteroposterior midline midbrain diameter, at the level of the superior colliculi on axial imaging demonstrating the “Mickey Mouse sign”, and loss of the lateral convex margin of the tegmentum of midbrain demonstrating the “Morning Glory sign” (C). Magnetic resonance parkinsonism index (MRPI) is calculated by multiplying the pons area to midbrain area ratio (D), in the midsagittal plane, by the middle cerebellar peduncle (F) width to superior cerebellar peduncle width ratio (E). Multiple system atrophy- parkinsonian type (MSA-P): Axial FLAIR (G),Proton Density (PD) (H) and Gradient Echo (GRE) (I) weighted MRI show linear region of high signal surrounding the lateral aspect of the putamen flatted demonstrating the “putaminal rim sign”. Cerebellar predominant type MSA (MSA-C). Midsagittal and axial T2-weighted (J,K) and PD-weighted MRI (L) show disproportionate atrophy of the cerebellum and pons, specially pontine tegmentum and middle cerebellar peduncle, with T2 hyperintensity in the pons forms a cross on axial images, representing selective degeneration of transverse pontocerebellar tracts and median pontine raphe (“hot cross bun sign”). Corticobasal degeneration. Right Parasagittal T1-weighted (M) and axial FLAIR-weighted (N) MRI images show asymmetric cortical atrophy of perirolandic gyri, most evident on the right.

Figure 2, included in this article, illustrates the radiological signs and the MRPI calculation described above.

## IRON-SENSITIVE MRI, NIGROSOME-1 AND DORSAL NIGRAL HYPERINTENSITY

The *substantia nigra* is a key structure for understanding the anatomical and functional changes that involve neurodegeneration in PD^21^. The *substantia nigra* pars compacta (SNc), located dorsally in the midbrain, contains dopaminergic neurons distributed in two different regions, from an immunohistochemical setting: a calbindin-rich matrix and poor-calbindin zones, called nigrosomes. There are five nigrosomes, and the largest, located dorsally in the *substantia nigra*, corresponds to nigrosome-1^9^ ^,^ ^22^.

Through high-field magnetic susceptibility-weighted imaging, the nigrosome-1 reveals itself as a hyperintense linear, “comma” or “wedge” shaped structure in the posterior third of the *substantia nigra*, labeled dorsal nigral hyperintensity^23^ ^,^ ^24^. 

**Figure 3.**
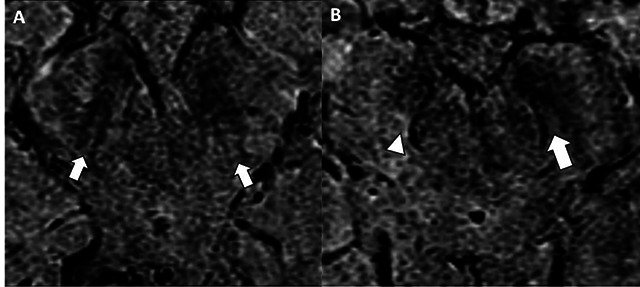
Nigrosome-1. Assessment of the substantia nigra using 3T high resolution susceptibility-weighted MR imaging at the level of nigrosome-1 in 2 different patients. The control subject (A) shows normal nigrosome-1 present bilaterally (arrow) and the PD patient (B) demonstrates right nigrosome-1 absent (arrowhead).

Medially, dorsal nigral hyperintensity is surrounded by low SWI signal intensity from the medial lemniscus, while laterally and anterior dorsal nigral hyperintensity is surrounded by a low signal from the pars compacta *substantia nigra*. Consequently, on axial imaging through high-field magnetic susceptibility-weighted imaging, nigrosome-1, and its surrounding structures resemble the morphology of a swallow's tail, called the “swallow-tail sign” appearance of the healthy nigrosome-1, as shown in Figure 3A ^23^ ^,^ ^24^.

Conversely, while it is unclear whether it is a cause or a consequence in pathogenesis, there is an iron overload in the *substantia nigra* in patients with PD^25^. A histopathological study shows a 31-35% increase in the total iron content of the parkinsonian *substantia nigra* when compared to healthy controls^25^ ^,^ ^26^.

Consequently, through high-field magnetic susceptibility sequences on MRI, due to iron overload in the context of nigrostriatal degeneration, loss of dorsal nigral hyperintensity and loss of the “swallow-tail sign” can be observed in PD patients, as shown in Figure 3B ^9^ ^,^ ^11^ ^,^ ^27^. 

Loss of dorsal nigral hyperintensity has emerged as a potential biomarker to differentiate PD patients from healthy controls^9^ ^,^ ^17^ ^,^ ^28^ ^,^ ^29^. A recent meta-analysis including ten studies, 364 PD and 264 control patients, demonstrated sensitivity and specificity of the absence of dorsolateral nigral hyperintensity to differentiate between the two groups greater than 90%^28^. However, the same study showed that the absence of DNH was also present in 89.4% of patients with AP disorders, probably reflecting the joint nigrostriatal degeneration of these conditions^28^. Moreover, two studies demonstrated that the absence of DNH could predict ipsilateral changes in neuroimaging of the dopamine transporter with sensitivity and specificity greater than 80%^30^ ^,^ ^31^.

Therefore, despite an emerging potential biomarker to demonstrate nigrostriatal neurodegeneration with apparently reasonable reproducibility to differentiate PD patients from healthy controls, high-field iron-sensitive images seem to have little accuracy for the differential diagnosis between neurodegenerative Parkinsonisms^9^ ^,^ ^28^.

In addition to its diagnostic value in PD, recent literature investigates the role of iron-sensitive MRI as a possible biomarker of disease progression through different imaging patterns depending on the stage of the disease^27^ ^,^ ^32^ ^,^ ^33^. A longitudinal study comparing neuroimaging findings in R2* relaxation imaging and quantitative susceptibility mapping (QSM) across different disease stages showed a significantly SNc faster increase on R2* in later-stage PD (>5 years of disease) when compared to early-stage PD (<1year) or middle-stage PD (<5 years)^34^.

When it comes to a potential biomarker during prodromal disease, a comparison among healthy controls, idiopathic rapid eye movement sleep behavior disorder (iRBD) patients, and PD patients through QSM demonstrated higher mean magnetic susceptibility values in the bilateral *substantia nigra* from iRBD patients compared to healthy controls. In contrast, mean magnetic susceptibility values were positively correlated with disease duration in the *substantia nigra* ^33^. Besides a potential diagnostic biomarker during the prodromal phase, such findings suggest that QSM can help monitor disease progression even in its earliest stages. Accordingly, PD patients had increased iron in the bilateral *substantia nigra*, globus pallidus, left red nucleus, and elevated iron levels in the bilateral *substantia nigra* compared with iRBD patients. This finding suggests the role of QSM as a biomarker of disease progression, which may be maintained after the phenoconversion from iRBD to PD^33^.

Despite the increasing availability of high-field scanners and the use of magnetic susceptibility sequences in the complementary investigation of suspected PD, with emphasis on DNH assessment, there is no definitive consensus on its use yet, and the lack of standardized imaging protocols, including spatial resolution and imaging planes, may limit their usefulness^9^.

## NEUROMELANIN-SENSITIVE MRI

Neuromelanin is an intracellular, dark, and insoluble pigment found in higher concentrations in catecholaminergic neurons, especially dopaminergic neurons of the *substantia nigra* and noradrenergic neurons of locus coeruleus^35^. Neuromelanin has the property of high affinity to chelate iron and bind neurotoxic metals that could promote neurodegeneration, and it appears to have antioxidant properties contributing to regulating the cellular oxidative stress, protecting endogenous dopamine^36^ ^,^ ^37^.

**Figure 4.**
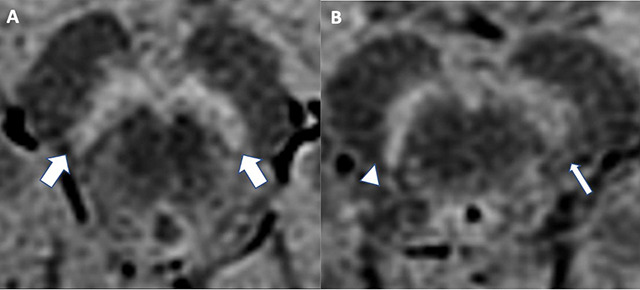
Neuromelanin. Assessment of the substantia nigra using 3T high resolution T1-weighted MR imaging in the midbrain structures in 2 different cases. The control subject (A) shows normal nigral hyperintensity present bilaterally (thick arrow) and the PD patient (B) demonstrates right loss of dorsolateral nigral hyperintensity (arrowhead) and left dorsolateral nigral hyperintensity reduced (thin arrow).

The neuromelanin-iron complex acts as a paramagnetic agent^37^ ^,^ ^38^. In this context, neuromelanin-sensitive MRI techniques have been improved in recent years: on T1-weighted fast spin-echo images at high-field MRI, brain regions containing melanin can be identified as areas of high signal intensity when compared to surrounding brain tissue (Figure 4A)^37^ ^-^ ^39^.

In PD, neuromelanin-containing neurons preferentially degenerate^40^. Consequently, through signal attenuation in regions where neurodegeneration occurs (Figure 4B), neuromelanin-sensitive MRI has emerged in several studies as a potential imaging biomarker to diagnose and track PD progression^27^ ^,^ ^35^ ^,^ ^38^ ^,^ ^39^.

In early PD patients, the lateral portion of the *substantia nigra* appears to be the topography where signal attenuation is most relevant^41^. Measurement of signal attenuation in the lateral portion of the *substantia nigra* demonstrated sensitivity and specificity greater than 70% and 80%, respectively, comparing early-stage PD patients with healthy controls^41^. Interestingly, the measurement of signal attenuation at the locus coeruleus has been shown to have greater sensitivity and specificity (82% sensitivity and 90% specificity), which suggests early neuronal depletion in the early disease stages and highlights the importance of emerging biomarkers in deepening the knowledge about the mechanisms that drive neurodegeneration in PD^39^ ^,^ ^41^.

On the other hand, the role of neuromelanin-sensitive MRI as a tool for the differential diagnosis between PD and AP presents less clear evidence, despite recent advances. In a prior study including healthy controls and early-onset parkinsonism patients, after a one-and-a-half year follow-up of PD, PSP, and MSA-P diagnosis, the signal intensity of the lateral, central, and medial parts of the SNc, the locus coeruleus, and the contrast ratios against adjacent white-matter structures were calculated. The lateral SNc contrast ratio was lower in the PD and MSA-P groups than in the PSP and control groups, while the contrast ratio of the locus was observed to be lower in the PD group than in the other groups^42^. In another recent study, the SNc estimated in neuromelanin-sensitive MRI was significantly smaller in PSP patients compared to PD patients and healthy controls^43^.

As an emerging neuroimaging biomarker, there is concern about assessing coherence and reproducibility with more well-established biomarkers such as dopamine transporter neuroimaging. The *substantia nigra* area on neuromelanin-sensitive MRI appears to be directly correlated with dopamine transporter density on SPECT neuroimaging, suggesting that neuromelanin-MRI may be a potential biomarker to quantify *substantia nigra* pathology and dopaminergic loss in PD^44^. 

From the same perspective as a potential biomarker of PD progression, through longitudinal follow-up, the *substantia nigra* volume and signal intensity on neuromelanin-MRI showed a more significant reduction with longer disease duration^38^. The levodopa equivalent daily dose (LEDD) in patients did not correlate with any *substantia nigra* MRI measurements, suggesting that dopaminergic medication did not modify neuromelanin-MRI signal changes^38^.

The recent literature suggests that neuromelanin-sensitive MRI is a potential biomarker for PD, but it still lacks standardized image processing and analysis protocols, which may limit its use in daily clinical practice^9^ ^,^ ^27^.

## DIFFUSION IMAGING

Diffusion-weighted imaging and diffusion tensor imaging might be a helpful tool to indirectly quantify the microstructural integrity through analysis of the overall displacement of water molecules, characterized as mean diffusivity, and the degree of displacement in space known as fractional anisotropy^12^. Briefly, degeneration of white matter tracts leads to an increase in mean diffusivity, while a decrease in fractional anisotropy is expected^12^. Consequently, analysis of mean diffusivity and fractional anisotropy in structures affected by neurodegeneration in PD has been a research target. 

Prior studies described a significant reduction in fractional anisotropy in the *substantia nigra* in PD patients compared to controls^17^ ^,^ ^45^ ^,^ ^46^. Such reduction was more pronounced in the caudal portion of the *substantia nigra*, which is congruent with the more intense neuronal loss in this structure as neurodegeneration progresses^45^ ^,^ ^46^. A reduction in fractional anisotropy was also observed in the anterior olfactory structures, in line with previous observations from olfactory disturbances in PD patients^12^ ^,^ ^17^ ^,^ ^47^. 

Literature data are conflicting: some studies report no fractional anisotropy or mean diffusivity significant differences between healthy controls and early PD patients^12^ ^,^ ^48^. One longitudinal study showed no significant differences, at the baseline, between healthy controls and PD patients. However, after a mean follow-up of 19 months, the PD patients showed a *substantia nigra* significant increased mean diffusivity and reduced fractional anisotropy. This change observed during follow-up analysis suggests that *substantia nigra* diffusion measure may be a valuable biomarker of PD progression^49^.

New image postprocessing methods, notably freewater imaging, also seem to have a promising role as potential new biomarkers^9^. Freewater in the posterior *substantia nigra* is elevated in PD patients compared to healthy controls. In addition, freewater level was correlated with disease duration, the severity of motor symptoms, and degree of dopaminergic loss on neuroimaging of the dopamine transporter, suggesting that it may be a valuable tool for diagnosing and monitoring disease progression^50^.

Hence, diffusion-weighted, tensor, and freewater imaging can also be valuable tools for differential diagnosis between PD and AP. A recent meta-analysis showed a 90% sensitivity and 93% specificity of diffusion-weighted MRI to differentiate MSA-P from PD through the analysis of putaminal diffusion, which is increased in patients with MSA-P^51^. More recently, a study proposed an approach involving diffusion-weighted imaging, free water postprocessing, in conjunction with automated analysis and machine learning algorithms, labeled automated imaging differentiation of parkinsonism (AID-P), as a practical and promising tool in differentiating PD from AP^52^. 

## DOPAMINE TRANSPORTER IMAGING

**Figure 5.**
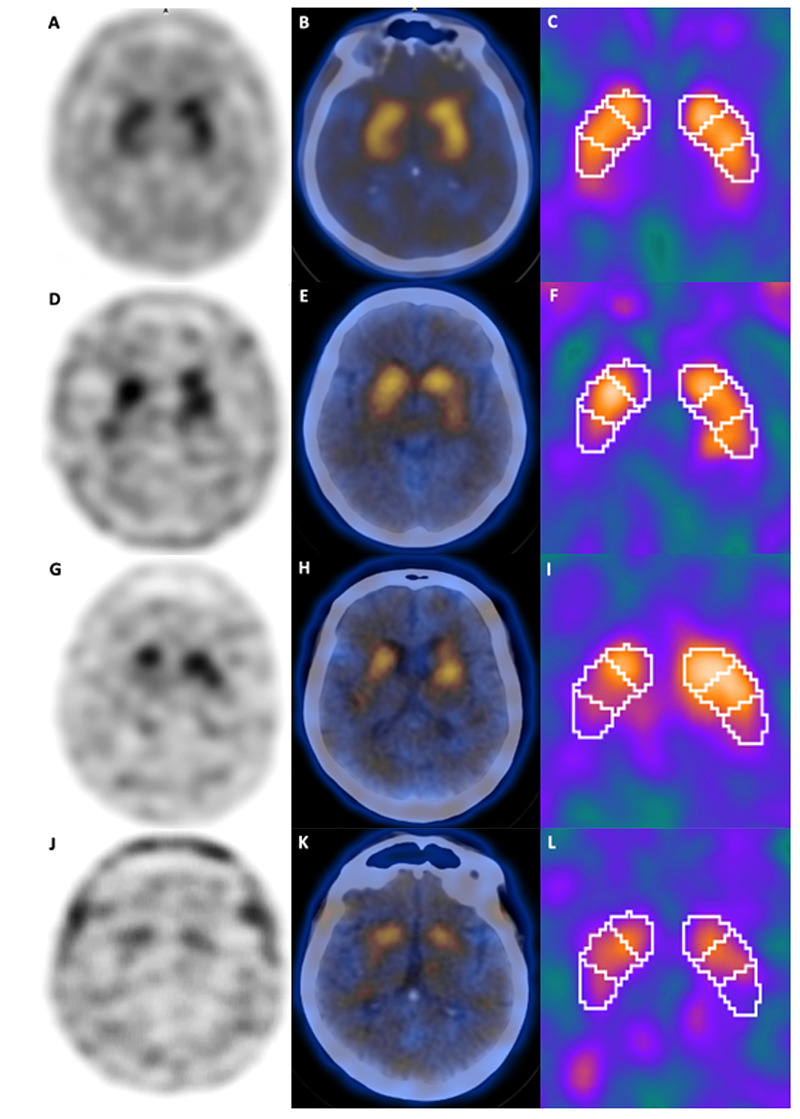
DAT-SPECT from healthy controls and from PD patients at different stages of the disease. A standard DAT-SPECT imaging appears as two intense symmetric “comma-shaped” regions of activity in the striatum (A, B,C). With the progression of PD, there is a decline in radiotracer uptake that follows a gradient from posterior to anterior structures, as shown through DAT-SPECT obtained during early (D,E,F), moderate (G,HI) and late (J,K,L) stage of PD.

In addition to MRI advances, dopamine transporter neuroimaging rises as an essential milestone in the diagnostic management of patients with parkinsonism or suspected PD. Presynaptic dopamine transporter (DAT) consists of a transmembrane sodium chloride-dependent protein expressed only in presynaptic dopaminergic cells, responsible for dopamine reuptake from the synaptic cleft^53^ ^,^ ^54^. The administration of radiotracers with high specificity for DAT combined with single-photon emission computed tomography (SPECT) imaging technique allows the assessment of DAT density at presynaptic terminals^53^. [123I]FP-CIT (123I-ioflupane) correspond to the most commonly used ligand^53^, although there are other radiotracers also with high specificity for DAT, such as [99mTc]TRODAT (frequently used in Brazil), [123I]β-CIT and [123I]IPT^53^ ^,^ ^55^. Standard DAT-SPECT imaging appears as two intense symmetric “comma-shaped” regions of activity in the striatum (Figure 5A, 5B, 5C). 

Due to neuronal loss in the nigrostriatal pathway occurring in neurodegenerative parkinsonisms, there is a reduction in the expression of DAT on presynaptic terminals, which leads to a reduction in radioligand striatal uptake in DAT-SPECT^53^ ^,^ ^56^. Decreased radiotracer binding, especially in the early stages of the disease, shows a rostrocaudal gradient pattern, with relative sparing of the caudate nucleus compared to the putamen (Figure 5D, 5E, 5F) ^53^ ^,^ ^56^. Loss in uptake also tends to be asymmetrical, as it is often more pronounced in the contralateral side to parkinsonism^17^ ^,^ ^53^ ^,^ ^56^. Unlike other neuroimaging biomarkers previously discussed, which are mostly restricted to the research environment, a normal DAT-SPECT has been incorporated as an absolute exclusion criterion in the 2015 MDS clinical diagnostic criteria for PD^5^.

Therefore, DAT-SPECT is a valuable tool in differentiating with high accuracy presynaptic neurodegenerative parkinsonisms from other clinical conditions, such as essential tremor and secondary parkinsonisms, such as vascular, psychogenic, or drug-induced parkinsonism^9^ ^,^ ^56^ ^,^ ^57^. Therefore, if DAT radiotracer binding is normal, the diagnosis of neurodegenerative parkinsonism becomes less likely^58^. DAT-SPECT, specifically 123I-ioflupane SPECT, commercially traded as DaTSCAN®, has been approved by the US Food and Drug Administration (FDA) and the European Medicines Agency as a complementary tool in the differential diagnosis between essential tremor and PD or other neurodegenerative parkinsonism related-tremor ^57^.

However, since both PD and AP are characterized by presynaptic involvement and nigrostriatal degeneration in their etiopathogenesis, the role of DAT-imaging in the differential diagnosis between the two conditions seems limited^53^ ^,^ ^56^ ^,^ ^58^. Some attempts to identify different patterns of ligand uptake among these conditions have been made, such as the recognition of more asymmetric uptake changes in patients with PD and corticobasal degeneration at a population level compared to patients with PSP and MSA^56^ ^,^ ^59^. Thus, on an individual level, DAT imaging does not appear to be a reliable tool in the discrimination of different causes of degenerative parkinsonism, and its use is not recommended for this purpose in routine clinical practice^53^ ^,^ ^56^ ^,^ ^60^.

The acronym SWEDD (scans without evidence for dopaminergic deficit) was coined after recognizing that some patients had normal DAT imaging, despite a presumed clinical diagnosis of PD^61^ ^,^ ^62^. As a recent review points out, patients with SWEDD form a heterogeneous group: most cases correspond to diverse medical conditions misdiagnosed as PD, such as essential tremor, dystonia, secondary or psychogenic parkinsonisms, depression with psychomotor slowness, and soft extrapyramidal signs of the elderly^62^. Conversely, a portion of SWEDD patients remained under the main hypothesis of PD, and some of them converted to altered DAT imaging during their follow-up, supporting the notion that an initial normal DAT-SPECT cannot permanently exclude early degenerative parkinsonism^62^. 

As the term SWEDD does not represent a single clinical entity, but only an absence of a dopaminergic imaging abnormality from a largely heterogeneous group of patients, some authors defend that this term should be abandoned^53^ ^,^ ^62^.

Finally, increasing data regarding dopamine transporter imaging has shown its role in the prodromal phase of PD. In patients with hyposmia, abnormal uptake on DAT-SPECT is a predictive factor of phenoconversion to PD, while in patients with iRBD, a DAT deficit identifies patients at short-term risk for synucleinopathy^63^ ^,^ ^64^. DAT imaging may also help to understand the heterogeneity of PD during the prodromal phase. Recent studies suggest two subtypes of prodromal PD according to the temporal and spatial pattern of alpha-synuclein progression: a body-first subtype, characterized by the early involvement of enteric autonomic nervous system and later progression to the central nervous system via the vagus nerve, and a brain-first subtype, characterized by the early brain involvement, with later progression to the brainstem and the peripheral autonomic nervous system. Through a multimodal approach, early alteration in DAT imaging helps to identify brain-first subtype prodromal disease patients^65^ ^,^ ^66^.

In conclusion, neuroimaging biomarkers in PD have made substantial progress in recent years with the advent of high-field MRI, improved sequences, and dopamine transporter ligands capable of assessing the integrity of the nigrostriatal pathway in vivo. 

Although some of these emerging biomarkers lack validation in the earlier stages of the disease, their role in clinical practice and diagnostic accuracy might increase with the future establishment of standardized image processing and analysis protocols, new forms of a multimodal approach, and machine-learning algorithms.
